# Endometrial cancer risk stratification using MRI radiomics: corroborating with choline metabolism

**DOI:** 10.1186/s40644-024-00756-x

**Published:** 2024-08-24

**Authors:** Yenpo Lin, Ren-Chin Wu, Yu-Chun Lin, Yen-Ling Huang, Chiao-Yun Lin, Chi-Jen Lo, Hsin-Ying Lu, Kuan-Ying Lu, Shang-Yueh Tsai, Ching-Yi Hsieh, Lan-Yan Yang, Mei-Ling Cheng, Angel Chao, Chyong-Huey Lai, Gigin Lin

**Affiliations:** 1 Department of Medical Imaging and Intervention, Chang Gung Memorial Hospital at Linkou, 5 Fuhsing St, Guishan, Taoyuan 33382 Taiwan; 2 Department of Medical Imaging and Radiological Sciences, Chang Gung University, Taoyuan, Taiwan; 3 Clinical Metabolomics Core and Imaging Core Laboratory, Institute for Radiological Research, Chang Gung Memorial Hospital and Chang Gung University, Taoyuan, Taiwan; 4 Department of Pathology, Chang Gung Memorial Hospital at Linkou, Taoyuan, Taiwan; 5 Department of Obstetrics and Gynecology and Gynecologic Cancer Research Center, Chang Gung Memorial Hospital at Linkou, Taoyuan, Taiwan; 6 Graduate Institute of Applied Physics, National Chengchi University, Taipei, Taiwan; 7 Research Center for Radiation Medicine, Chang Gung University, Taoyuan, Taiwan; 8 Clinical Trial Center, Chang Gung Memorial Hospital at Linkou, Taoyuan, Taiwan; 9 Division of Clinical Research, Taichung Veterans General Hospital, Taichung, Taiwan; 10 Department of Biomedical Sciences, College of Medicine, Chang Gung University, Taoyuan, Taiwan

**Keywords:** Endometrial carcinoma, Diffusion-weighted imaging, Diagnostic accuracy, Magnetic resonance imaging, Magnetic resonance spectroscopy

## Abstract

**Background and purpose:**

Radiomics offers little explainability. This study aims to develop a radiomics model (Rad-Score) using diffusion-weighted imaging (DWI) to predict high-risk patients for nodal metastasis or recurrence in endometrial cancer (EC) and corroborate with choline metabolism.

**Materials and methods:**

From August 2015 to July 2018, 356 EC patients were enrolled. Rad-Score was developed using LASSO regression in a training cohort (*n* = 287) and validated in an independent test cohort (*n* = 69). MR spectroscopy (MRS) was also used in 230 patients. Nuclear MRS measured choline metabolites in 70 tissue samples. The performance was compared against European Society for Medical Oncology (ESMO) risk groups. A *P* < .05 denoted statistical significance.

**Results:**

Rad-Score achieved 71.1% accuracy in the training and 71.0% in the testing cohorts. Incorporating clinical parameters of age, tumor type, size, and grade, Rad-Signature reached accuracies of 73.2% in training and 75.4% in testing cohorts, closely matching the performance to the post-operatively based ESMO's 70.7% and 78.3%. Rad-Score was significantly associated with increased total choline levels on MRS (*P* = .034) and tissue levels (*P* = .019).

**Conclusions:**

Development of a preoperative radiomics risk score, comparable to ESMO clinical standard and associated with altered choline metabolism, shows translational relevance for radiomics in high-risk EC patients.

**Trial registration:**

This study was registered in ClinicalTrials.gov on 2015–08-01 with Identifier NCT02528864.

**Supplementary Information:**

The online version contains supplementary material available at 10.1186/s40644-024-00756-x.

## Introduction

Endometrial cancer (EC) is the most prevalent malignancy in the female reproductive system in the United States [1]. According to European Society for Medical Oncology (ESMO) guidelines, EC can be categorized into risk groups based on tumor type, tumor grade, extent of myometrial invasion, lymphovascular space invasion (LVSI) and molecular subgroups [2]. However, preoperative evaluation of LVSI remains challenging. While surgery is the primary staging and treatment method, it's crucial to avoid unnecessary systemic pelvic or paraaortic lymphadenectomy in low-risk patients while ensuring comprehensive treatment for higher-risk groups to enhance survival outcomes [3].

Magnetic resonance imaging (MRI), particularly with the inclusion of diffusion-weighted imaging (DWI), is recommended for preoperative assessment of EC, especially for evaluating myometrial invasion [4]. Radiomics is an emerging imaging tool that uses machine learning to extract quantitative imaging features for noninvasive tumor characterization and predicting tumor behavior [5]. Several attempts have been made in EC to develop a prognostic radiomic model [6–9]. However, the reproducibility and clinical impact of radiomic models in EC are still under debate [10].

Proton MR Spectroscopy (^1^H MRS) allows in vivo detection of metabolic and molecular compositions, frequently identifying an elevated total choline (tCho) signal in various cancers [11]. With high-resolution ^1^H MRS, it is possible to differentiate individual choline components, including phosphocholine (PC), glycerophosphocholine (GPC), and free choline (Cho). The presence of deregulated choline biochemistry on preoperative MRS can provide a biochemical basis for risk stratification [12].

This study aims to develop a radiomics risk model using routine DWI MRI to predict the high-risk group for nodal metastasis or cancer recurrence in EC and corroborate with underlying choline metabolic pathways. We hypothesized that the DWI radiomics risk model will improve the accuracy for identifying high-risk EC patients with nodal metastasis or cancer recurrence compared to ESMO risk groups.

## Materials and methods

### Patient cohort

This study complied with the Transparent Reporting of a Multivariable Prediction Model for Individual Prognosis or Diagnosis Statement and in accordance with the Declaration of Helsinki. The institutional review board approved this prospective study (NCT02528864) conducted in a tertiary referral center with a dedicated gynecology oncology interdisciplinary team. Informed consent was obtained. Between August 2015 and July 2018, a consecutive cohort of 550 female patients underwent surgical intervention for EC. Inclusion criteria included (1) female aged 20–80, (2) clinical suspicion of endometrial malignancy for MR pretreatment staging, (3) the surgical intervention included, at a minimum, a hysterectomy, and may or may not encompass a bilateral adnexectomy or lymphadenectomy. Exclusion criteria included (1) contraindications to MR scanning, such as claustrophobia, cardiac pacemaker, and metal implants in the field of view. (2) lesion size < 1 cm^3^, (3) Noncompliant to treatment or not accessible for follow-up, (4) suboptimal MR imaging quality, and (5) pathology other than endometrial carcinoma, such as uterine sarcoma or neuroendocrine tumor. Data collection was planned before the MR imaging acquisition. Additional tumor tissue samples were gathered postoperatively to facilitate further radiometabolic analysis, adhering to an extra inclusion criterion mandating the availability of metabolomic data from the cellular tumor.

### Imaging protocol: DWI and MR spectroscopy

Preoperative MRI of the enrolled patients were performed on a 3 Tesla MR system (Skyra, Siemens, Erlangen, Germany) and both spine and body-phased array coils to image the entire pelvis in a supine position. Utilizing an external coil instead of an endovaginal coil is primarily motivated by reducing invasiveness and facilitating MRS incorporation into routine pelvic exams. No premedication was administered, and minimal breathing was maintained during the scan. To ensure reproducibility and robustness, a phantom with two water tubes and two gelatin tubes of varying concentrations (1% and 3%) embedded in iced water was used to test DWI reproducibility. Triplane localizer 1D MRS with point-resolved spectroscopy (PRESS) was used to collect data from a volume of interest measuring 12 × 12 × 12 mm^3^, prescribed by gynecological radiologists (Y-L.H. or G.L.), wholly placed within the endometrial tumor (high signal intensity area on high-b-value DW and high signal intensity on T1-weighted, fat-saturated post-contrast enhanced MRI; free of hemorrhage, necrosis or large arteries judging from T1- and T2-weighted images. MRS was conducted based on the interpreting radiologist's judgment to ascertain the feasibility of placing an adequate region of interest (ROI). LCModel software (v. 6.3–0 K; Provencher, Ontario, CA, Canada) was used to analyze the data, and resonance amplitudes for choline were integrated using the corresponding water signals as a reference. The Cramer-Rao lower bound (CRLB) value was calculated to estimate the error in metabolite quantification, and MR spectra were excluded if the CRLB exceeded 20% for choline. The imaging parameters used are provided in the supplementary material.

### Image processing and segmentation

The study used a monoexponential decay model to generate ADC maps with a b value of 0 and 1000 s/mm^2^. The MR images were independently interpreted by two radiologists with 6 and 10 years of experience in gynecological radiology (Y.L. and Y-L.H.), and discrepancies were resolved by a third reader with 20 years of experience (G.L.) for consensus. An in-house developed software based on Matlab was used to draw the ROI by the first radiologist (Y.L.) around the tumor on the ADC map with reference to the high b-value DW and T2-weighted images to delineate the whole tumor volume and avoid contaminating the adjacent normal endometrium or areas of fluid.

### Radiomics analysis

**Fig. 1:**
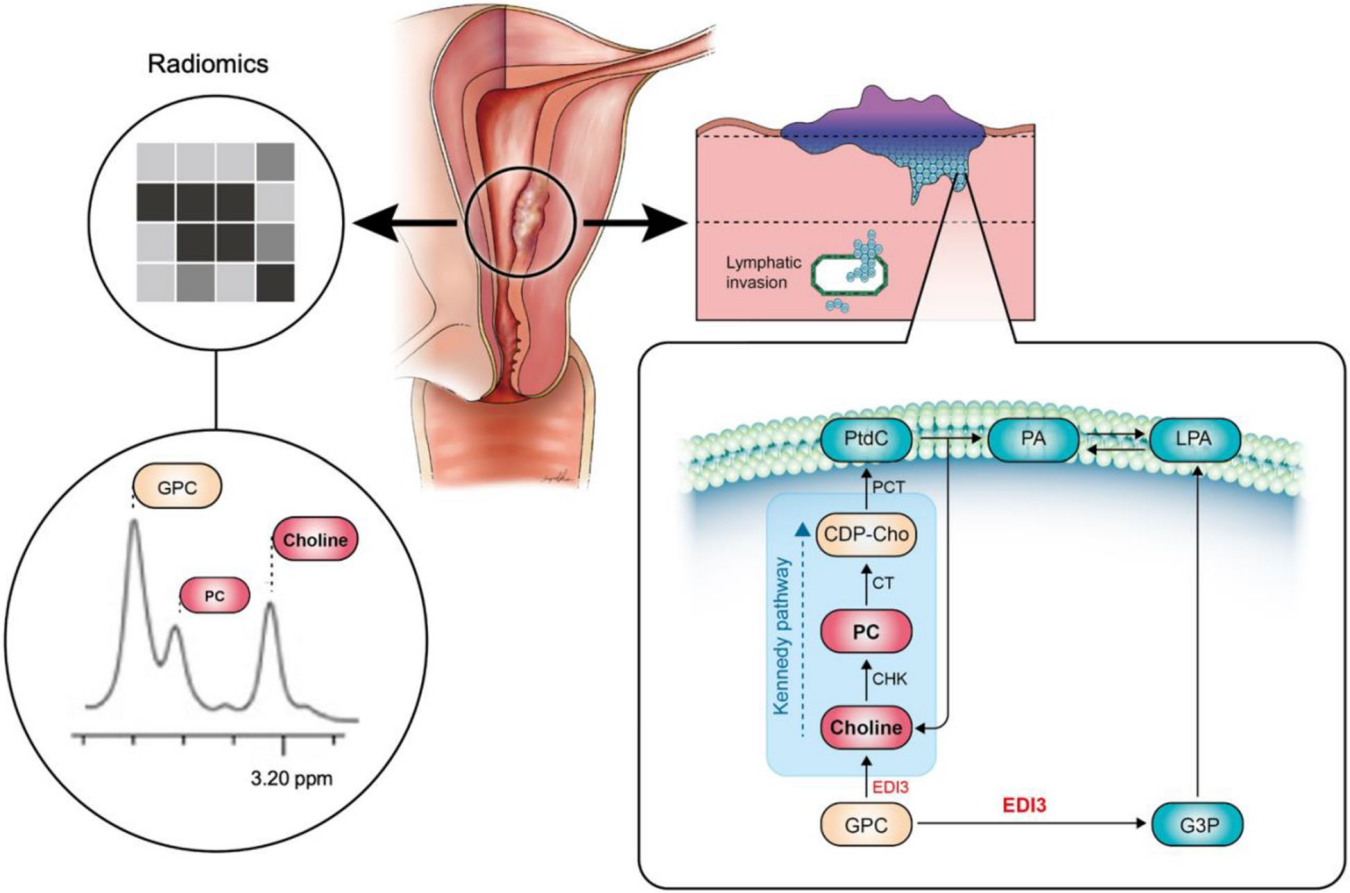
The framework of the study design (WIP)

MR images were normalized for signal intensities. First-order and 3D shape-based order features were extracted for radiomics analysis. Statistics of the median, standard deviation, skewness, and kurtosis were calculated from the feature responses of all voxels within the ROI. For feature selection, we employed the Least Absolute Shrinkage and Selection Operator (LASSO) logistic regression model to eliminate redundant features while retaining those most relevant to the outcome for building a radiomic score model, Rad-Score. The model’s performance was evaluated using the Youden Index to determine the optimal cutoff value for predicting recurrence. A combined model, Rad-Signature, was also constructed with the Rad-Score model and selected preoperative clinical parameters such as age, histopathology (endometrioid or non-endometrioid), and tumor grade. Other ESMO risk parameters such as LVSI, myometrial invasion depth, or lymph node status required surgical histopathological verification and thus were not chosen to build the Rad-Signature. The models were tested in an independent dataset. The framework of this study is illustrated in Fig. 1. Details can be found in the supplementary material.

### Histopathology and clinical data

The study used histopathology from surgical hysterectomy specimens as the reference standard for evaluating endometrial tumors. Histopathology was evaluated for tumor type, grade, LVSI, pathological staging. Additional immunohistochemical studies were performed if the primary site of the tumor was uncertain. The surgical strategy for lymph node (LN) evaluation largely rested on the operating surgeons' discretion, aligned with the risk stratification of ESMO guideline, including no LN dissection, sentinel LN biopsy and pelvic and paraaortic lymphadenectomy. Clinical parameters, including age, tumor size, final cancer stage, lymph node metastasis, recurrence, and treatments, were also recorded. The patients were stratified into risk groups according to ESMO guidelines.

### Outcome measurement

All patients underwent standard surgical treatment. Patients with proven lymph node metastasis at surgical histopathology or cancer recurrence were classified as high-risk group, based on pathology documentation, and confirmed by at least two imaging modalities. Patients lost to follow-up, non-cancer-related deaths, and those alive at the end of the follow-up period were considered censored observations. Patients with persistent disease were regarded as having relapsed on the first day of completing standard primary therapy.

### Statistical analysis

The data was analyzed using standard statistical methods on MedCalc for Windows, Version 20.218. The Shapiro–Wilk test was used to check the normality of the data. The Student’s t-test was used to examine differences in radiomic features between high-risk and low-risk groups. To address the class imbalance in the present study, we increased the weight of the high-risk class (lymph node metastasis or recurrence) by a factor of 4. Correlations between various variables were evaluated using Pearson’s correlation analysis. Sensitivity, specificity, accuracy, positive predictive value (PPV), and negative predictive value (NPV) were calculated. Variables significant in the univariate analysis were subsequently entered into a multivariate logistic analysis, and their odds ratios (OR) and 95% confidence intervals (CI) were calculated. Bootstrap resampling was performed randomly to construct new data sets, followed by Cox regression analysis repeated 1,000 times. All tests were two-sided, and *P* < 0.05 was considered statistically significant.

### Data availability

The data generated in this study are available upon request from the corresponding author.

## Results

### Patient cohort

**Fig. 2:**
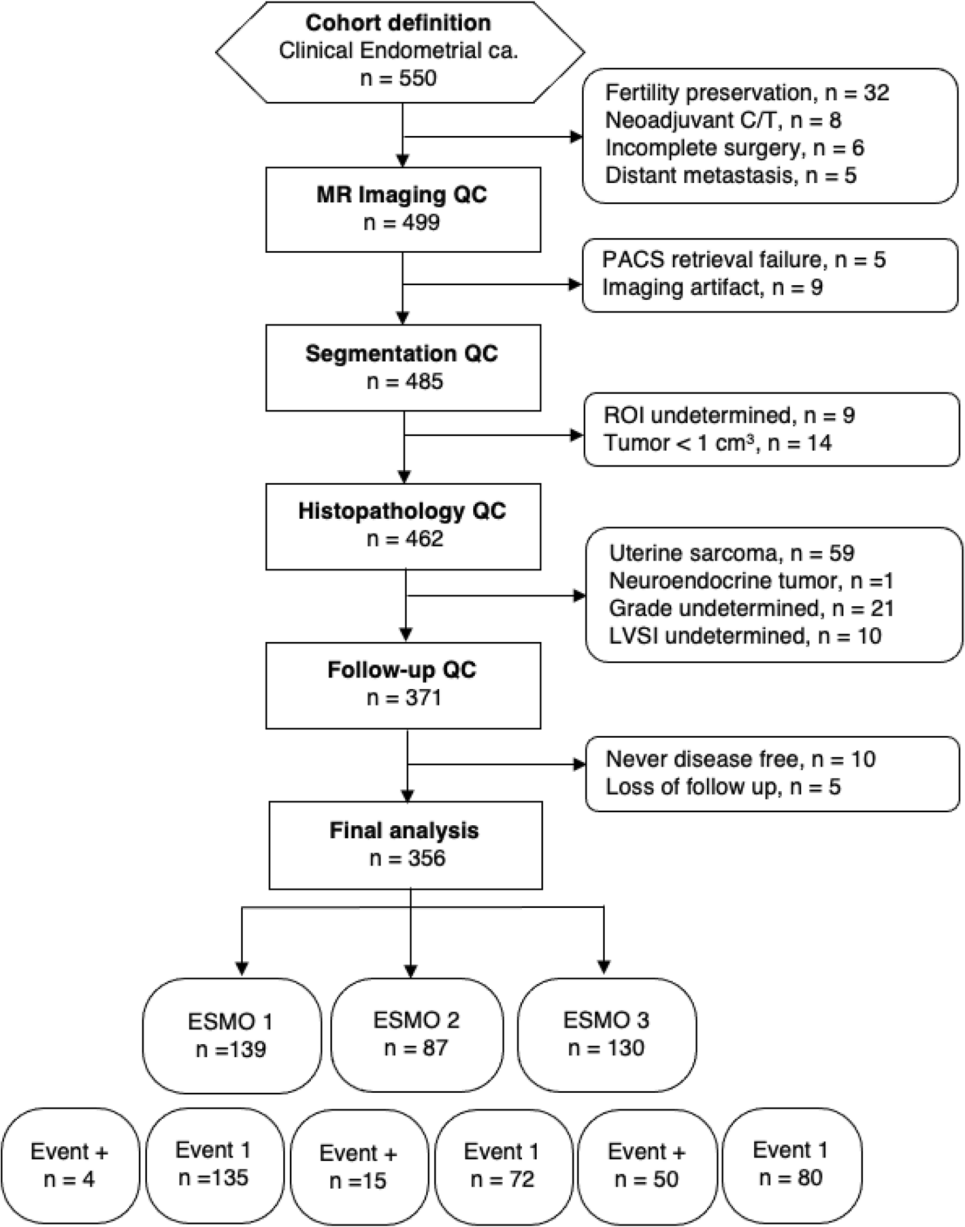
Flow diagram of the study cohort

Out of the 550 patients initially considered, 194 were excluded based on criteria detailed in Fig. 2, which presents a flow diagram illustrating the composition of the study cohort. Finally, 356 patients were eligible for analysis. The demographic data of the study population are listed in Table 1. There was no significant demographic difference between the training and testing cohorts. Further, MRS was performed in 240 of them, and 10 patients were excluded due to CRLB larger than 20%. For the tissue sample analysis, 70 EC tumor tissue samples with corresponding metabolomics data were investigated on a nuclear magnetic resonance (NMR) spectrometry platform.

**Table 1:** Clinical and demographic data of the study population

Variable	All	Training set	Testing set	*P* values
Patients number	356	287	69	
Age (years, range)	53 (25–88)	53 (25–88)	53 (26–76)	.871
Tumor pathologic size (mm, range)	36 (1–166)	36 (1–163)	35 (2–166)	.807
Histopathology				.767
Endometrioid	287 (80.6%)	230 (80.1%)	57 (82.6%)	
Non-endometrioid	69 (19.4%)	57 (19.9%)	12 (17.4%)	
Tumor grade				.527
1	171 (48.1%)	135 (47.0%)	36 (52.2%)	
2	112 (31.5%)	91 (31.7%)	21 (30.4%)	
3	73 (20.5%)	61 (21.3%)	12 (17.4%)	
LVSI				.654
None	278 (78.1%)	226 (78.7%)	52 (75.4%)	
Present	78 (21.9%)	61 (21.3%)	17 (24.6%)	
FIGO stage				.603
1A	239 (67.1%)	195 (67.9%)	44 (63.8%)	
1B	63 (17.7%)	51 (17.8%)	12 (17.4%)	
2	30 (8.4%)	24 (8.4%)	6 (8.7%)	
3A	16 (4.5%)	11 (3.8%)	5 (7.2%)	
3B	6 (1.7%)	4 (1.4%)	2 (2.9%)	
4	2 (0.6%)	2 (0.7%)	0 (0.0%)	
ESMO risk groups				1.000
Low	139 (39.0%)	110 (38.3%)	29 (42.0%)	
Intermediate	87 (24.4%)	72 (25.1%)	15 (21.7%)	
High-intermediate and high	130 (36.5%)	105 (36.6%)	25 (36.2%)	

Unless otherwise indicated, data are number of variables, with their percentage in parentheses

*EC* endometrioid carcinoma, *ESMO* European Society for Medical Oncology, *FIGO* International Federation of Gynecology and Obstetrics, *LVSI* lymphovascular invasion

Among the 356 patients studied, lymph node evaluation was not performed on 39 individuals. Sentinel lymph node biopsy was conducted on 33 patients, while the remaining 284 underwent either pelvic or systemic lymphadenectomy. The median follow-up time was 41 months (1–108 months) for surviving patients. Of the 356 women analyzed, 287 (80.6%) were disease-free at the last follow-up.

There were 69 patients in the high-risk group of this study due to either having lymph node metastasis at surgery or cancer recurrence during follow-up. There were 30 patients with nodal metastases at surgery. Of the 39 patients with recurrence, 16 had distant failure alone (five lungs, three peritoneum, one distant node, and seven having multiple sites), 3 had regional node recurrence alone, and 7 had local or regional failure alone. The remaining 13 patients had more than one site of failure: eleven local–regional-distant, and two regional-regional node-distant. At least two imaging modalities confirmed recurrences in all cases.

### Building Rad-Score model building and performance

**Fig. 3:**
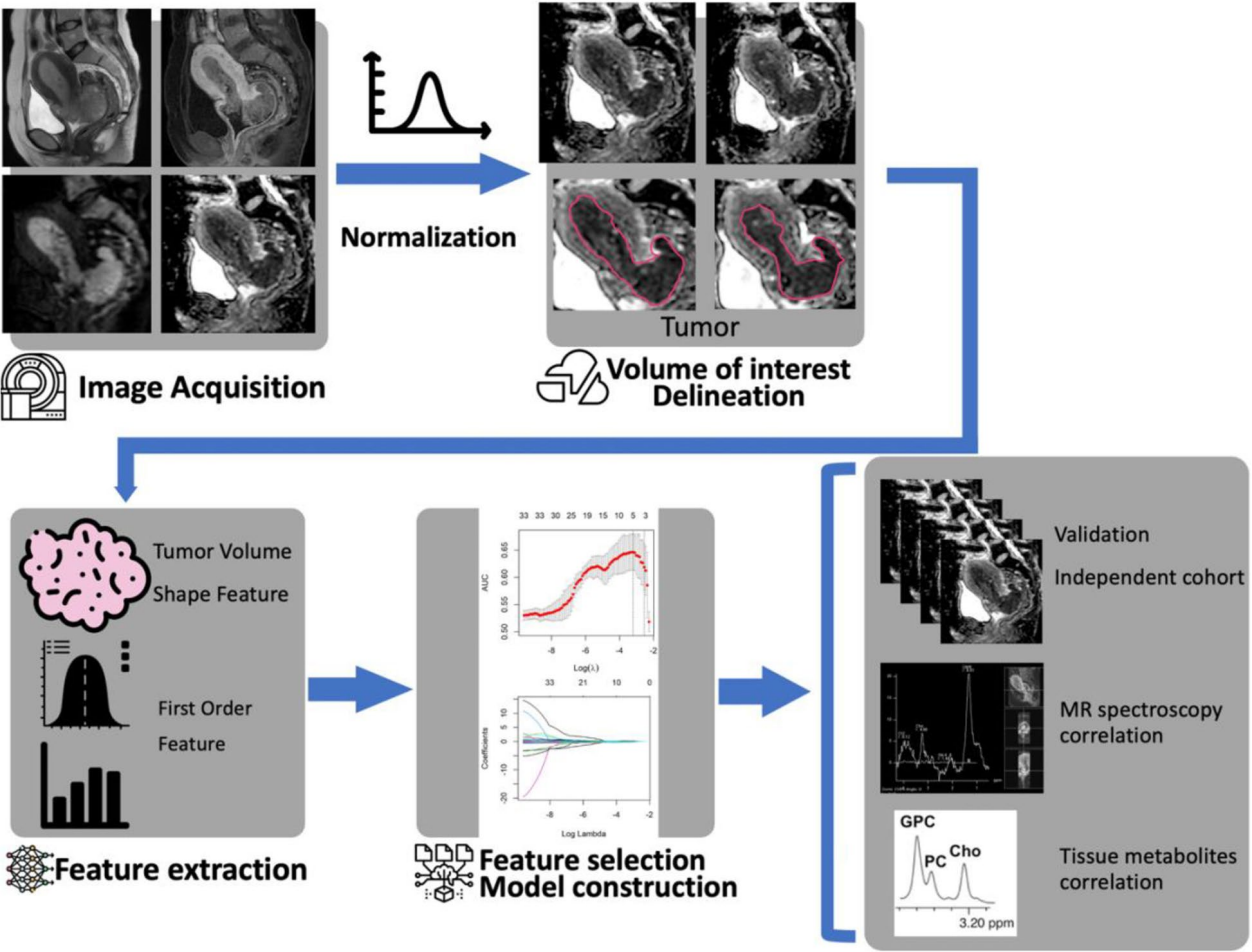
Rad-Score pipeline

The study confirmed DWI's reproducibility through using Bland–Altman plots in the first ten subjects. In the training cohort, 105 radiomic features were extracted from each segmented tumor volume on axial DWI, out of which 36 features exhibited statistical significance in the Student’s t-test between the recurrence and non-recurrence groups. These features encompass both first-order and shape-related attributes (Supplementary Table 1). After feature pruning by the LASSO regression method, the radiomics model (Rad-Score) was built from 17 3D DWI features and included first-order and shape features, as shown in Fig. 3. In the context of patient characteristics and treatment history, factors such as older age at diagnosis, larger tumor size, higher histological grade, presence of LVSI, higher FIGO stage, advanced ESMO group classification, and the absence of adjuvant radiation therapy or chemotherapy were found to be significantly correlated with classification into the high-risk group (*P* < 0.001 for all parameters), as tabulated in Table 2.

**Table 2:** Clinical and demographic data between risk groups predicted by Rad-Score

Variable	Rad-Score high risk	Rad-Score low risk	*P* values
Patients number	124	232	
Age (years, range)	56 (29–88)	51 (25–83)	< .001*
Tumor pathologic size (mm, range)	48 (3–163)	29 (1–166)	< .001*
Histopathology			.329
Endometrioid	96 (77.4%)	191 (82.3%)	
Non-endometrioid	28 (22.6%)	41 (17.7%)	
Tumor grade			< .001*
1	29 (23.4%)	142 (61.2%)	
2	55 (44.4%)	57 (24.6%)	
3	40 (32.3%)	33 (14.2%)	
LVSI			< .001*
None	81 (65.3%)	197 (84.9%)	
Present	43 (34.7%)	35 (15.1%)	
FIGO stage			< .001*
1A	52 (41.9%)	187 (80.6%)	
1B	39 (31.5%)	24 (10.3%)	
2	20 (16.1%)	10 (4.3%)	
3A	8 (6.5%)	8 (3.4%)	
3B	3 (2.4%)	3 (1.3%)	
4	2 (1.6%)	0 (0.0%)	
ESMO risk groups			< .001*
Low	20 (16.1%)	119 (51.3%)	
Intermediate	32 (25.8%)	55 (23.7%)	
High-intermediate and high	72 (58.1%)	58 (25.0%)	

Unless otherwise indicated, data are number of variables, with their percentage in parentheses

*EC* endometrioid carcinoma, *ESMO* European Society for Medical Oncology, *FIGO* International Federation of Gynecology and Obstetrics, *LVSI* lymphovascular invasion

^*^represents statistical significance

Applying a probability threshold greater than 0.5, the Rad-Score model achieved an accuracy of 71.1% (95% CI: 65.5%-76.3%) within the training cohort to distinguish high-risk patients. In the testing cohort, the model maintained a comparable accuracy of 71.0% (95% CI: 58.8%-81.3%), as detailed in Table 3. Additionally, we examined other clinical parameters such as age, tumor grade, and histopathology to discern differences between the low-risk and high-risk groups as classified by the Rad-Score, with these findings presented in Table 2.

**Table 3:** Performance of Rad-Score and Rad-Signature in the training and testing data compared to ESMO

	Sensitivity	Specificity	Accuracy	PPV	NPV
Training set					
Rad-Score	65.5% (51.4%-77.8%)	72.4% (66.2%-78.1%)	71.1% (65.5%-76.3%)	36.0% (26.6%-46.2%)	89.8% (84.6%-93.8%)
Rad-Signature	85.5% (73.3%-93.5%)	70.3% (63.9%-76.1%)	73.2% (67.6%-78.2%)	40.5% (31.5%-50.0%)	95.3% (91.0%-98.0%)
ESMO risk group	69.1% (55.2%-80.9%)	71.1% (64.8%-76.9%)	70.7% (65.1%-75.9%)	36.2% (27.0%-46.1%)	90.7% (85.5%-94.5%)
Testing set					
Rad-Score	64.3% (35.1%-87.2%)	72.7% (59.0%-83.9%)	71.0% (58.8%-81.3%)	37.5% (18.8%-59.4%)	88.9% (75.9%-96.3%)
Rad-Signature	78.6% (49.2%-95.3%)	74.5% (61.0%-85.3%)	75.4% (63.5%-84.9%)	44.0% (24.4%-65.1%)	93.2% (81.3%-98.6%)
ESMO risk group	85.7% (57.2%-98.2%)	76.4% (63.0%-86.8%)	78.3% (66.7%-87.3%)	48.0% (27.8%-68.7%)	95.5% (84.5%-99.4%)

The numbers in parentheses represent the 95% confidence interval

*ESMO* European Society for Medical Oncology, *PPV* positive predictive value, *NPV* negative predictive value

### Performance of Rad-Signature model

The assessment of radiomics' clinical utility has been further enhanced by introducing a combined model, Rad-Signature. This model integrates the Rad-Score model with clinical parameters, including age at diagnosis, clinical tumor size (cut-off, 20 mm), histopathologic type (endometrioid versus non-endometrioid), and histological grade (grade 1 and 2 vs. 3). Rad-Signature achieved a slightly better discriminative ability than the Rad-Score model with an accuracy of 73.2% (95% CI: 67.6%-78.2%) in the training cohort and an accuracy of 75.4% in the testing cohort (95% CI: 63.5%-84.9%) (Table 3).

### Comparison with ESMO standard of care score

The diagnostic efficacy of the radiomics models was compared to the standard of care, specifically the ESMO risk groups, distinguishing between high-risk versus low- and intermediate-risk groups. Both the Rad-Score and Rad-Signature models exhibited comparable diagnostic performance with the ESMO score within the training cohorts (accuracy: 71.1%, 73.2%, 70.7%, respectively). However, in testing cohorts, the ESMO score outperformed both models, achieving a superior accuracy of 78.3% versus 75.4% for the Rad-Signature and 71.0% for the Rad-Score (Table 3).

### Correlation of the Rad-Score model with MRS

The study deployed MRS in a subset of 230 patients to examine the association between specific radiomic features and underlying biological processes. There were 14 distinct radiomic features from the Rad-Score model that exhibited significant correlations with elevated total choline levels in the endometrium as determined by ^1^H MR spectroscopy; these features included both first-order statistics and shape descriptors (all *P* < 0.05), as listed in Table S2. Among the clinical parameters, the age at diagnosis showed a significant correlation with increased endometrial total choline levels on ^1^H MR spectroscopy (*P* = 0.011). Furthermore, the Rad-Score model was associated with heightened levels in GPC as measured by MRS (Pearson correlation coefficient [r] = 0.140, *P* = 0.034).

### Tissue choline NMR analysis

Out of 230 patients with MRS, 70 tissue samples from surgical hysterectomy were obtained to analyze GPC, PC, glycerol-3-phosphate (G3P), and free choline on the NMR spectrometry platform. The Rad-Score model was significantly correlated with changes of G3P level in tissue samples (*P* = 0.019). A few individual features also significantly correlated with changes of levels in G3P, choline, and PC (Table S2).

## Discussion

The study evaluated radiomics models from preoperative DWI in 356 EC patients, focusing on lymph node metastasis detected in surgery and cancer recurrence in the follow-up. The Rad-Score model, based solely on radiomics, reached a 71% accuracy in both training and testing cohorts. By integrating clinical parameters, the Rad-Signature model improved to accuracies of 73.2% and 75.4% in training and testing cohort, respectively. Additionally, radiomic features significantly correlated with increased total choline levels on MRS. While both models were comparable to the ESMO standard of care score within the training cohorts, they did not reach the ESMO score's 78.3% accuracy level in the testing cohorts.

A recent meta-analysis has reinforced the value of pre-operative MRI radiomics models for risk stratification in EC [13]. Among various models, a 3D radiomics approach stands out for matching or even surpassing the performance of experienced radiologists [14], highlighting its potential as a reliable tool. The present study employed 3D radiomics features derived from DWI, recognizing its significant role in the risk stratification of EC [15]. While prior studies had shown that ADC values could effectively predict high-risk disease, [16, 17] combining 3D DWI features enabled a more detailed prediction of myometrial invasion, LVSI and tumor grade [18]. Our study yielded comparable results, yet the Rad-Signature model outperformed the radiomics-only Rad-Score model, achieving an accuracy of 75.4%, closely matching the performance of the ESMO standard at 78.3%. This aligns with findings from a recent review, which concluded that radiomics models incorporating both clinical and radiomic features outperform those based solely on radiomic features [19].

The predictive model for lymph node metastasis in EC, utilizing MR radiomic features and clinical parameters, has demonstrated effective discrimination ability, particularly in normal-sized lymph nodes [20, 21]. While our model’s accuracy of 75.4% is not the highest reported, it falls within the range of performance observed in similar studies. For instance, a recent two-center study investigating pre-operative risk factors for EC reported accuracies ranging from 75 to 86% across multiple factors [14]. Additionally, another study evaluating a whole-lesion T2w-derived radiomics model for risk stratification achieved accuracies of 0.71 and 0.72 in the training and testing sets, respectively [9]. Furthermore, the Rad-Signature model in our study, based on preoperative parameters, achieved an excellent NPV of 95.3% in the training cohort, albeit slightly lower than the post-operatively based ESMO classification in the independent test cohort. Nonetheless, the Rad-Signature model's utility in identifying low-risk patients who may not benefit from lymphadenectomy is noteworthy. Given that EC management hinges on risk stratification [22], a fully automated radiomics pipeline utilizing pre-operative MRI data could be a future direction in integrating risk stratification into the clinical pathway more effectively [23].

The study further explored the biochemical processes underlying radiomics to enhance the model's explainability. By examining the correlation between radiomics-identified high-risk patients with elevated total choline levels on MRS, the radiomics model could potentially correlate with cell membrane choline metabolism. Regarding the correlation of DWI and biomarkers, a prior study found that patients with high Ki-67 expression had significantly lower mean ADC values than those with low Ki-67 expression [24]. MRS was employed in our study as abnormal choline metabolism is a characteristic cancer marker [11]. An elevated total choline (tCho) signal is detectable by MRS in all tested cancers, as well as in various stages of EC [11, 25, 26]. Specifically, a high tCho/Water ratio and choline peak could differentiate EC from benign endometrium, differentiating type II from type I EC and identifying high-grade tumors [12, 27, 28].

**Fig. 4:**
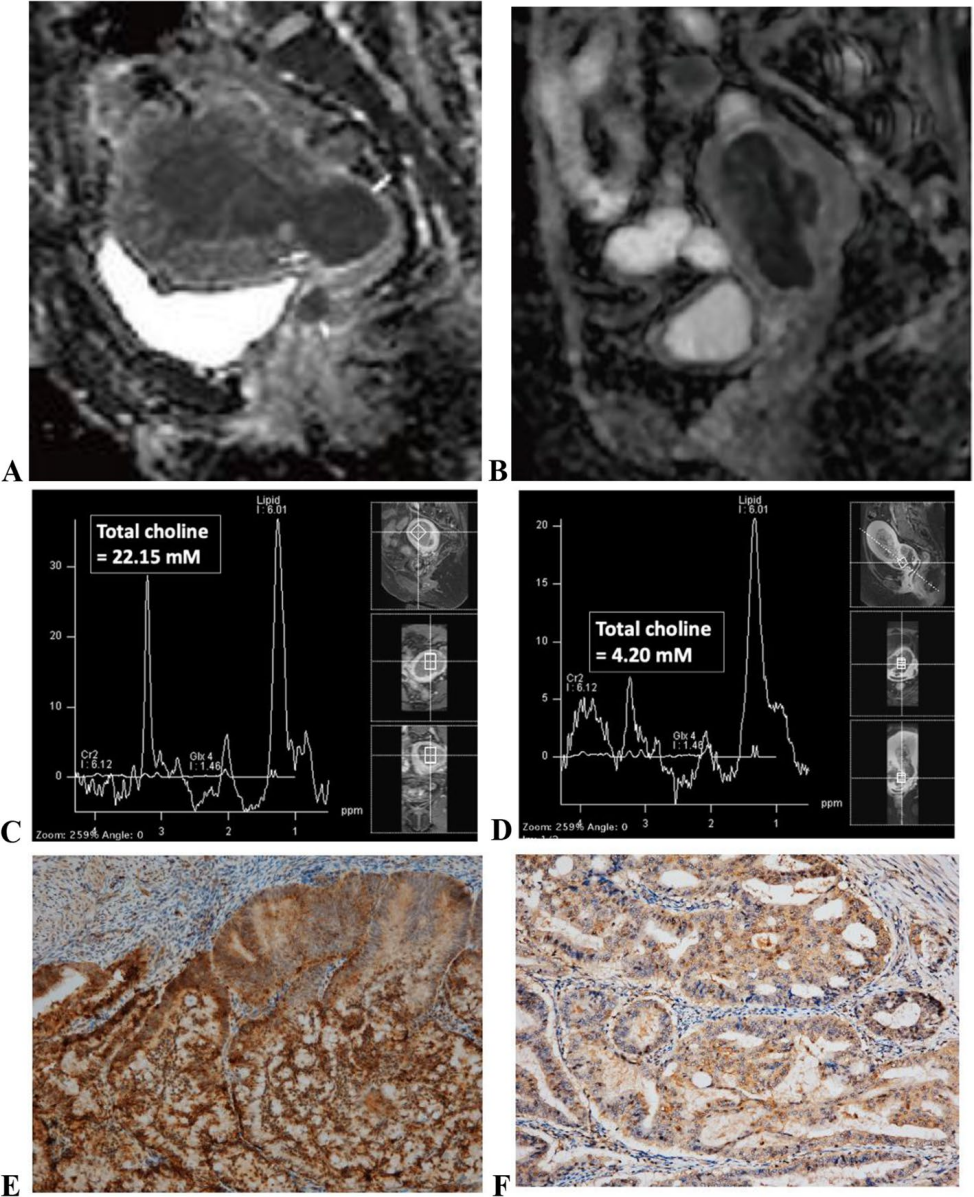
Comparative imaging and analysis of two different patients with grade 3 endometrial cancer using radiomics from ADC map, MR spectroscopy (MRS), and electron microscopy.

Additionally, we analyzed a subset of 70 tissue samples from hysterectomies for various metabolites using the NMR spectrometry platform. The Rad-Score model exhibited a significant correlation with G3P levels. Correlations between individual radiomic features and G3P, choline, and phosphocholine levels were also observed. The alteration of choline phospholipid metabolism is primarily due to choline kinase alpha overexpression and a hyperactivated deacylation pathway, leading to a 70% increase in phosphocholine levels [29]. Furthermore, endometrial carcinoma differential 3, a key enzyme in choline metabolism, increases levels of active protein kinase C by breaking down glycerophosphocholine. Its overexpression is commonly observed in endometrial and ovarian cancer patients, which correlates with a higher risk of metastasis and decreased survival rates [30, 31] (Fig. 4).

This study had some limitations. First, MRS data were not included in the radiomics model. This was primarily due to the absence of standardized protocols for MRS data acquisition and analysis across various imaging centers, which could result in inconsistent and unreliable data. Secondly, the study was conducted at a single center using a dedicated MR scanner. The lack of external validation and data from a single center may limit the generalizability of our findings. Future studies incorporating multicenter data are recommended to further validate the robustness of the radiomics model. Furthermore, while we excluded tumors with a volume less than 1.5 cm to avoid partial volume effects, extreme ADCs may still result from DW imaging and ADC map misregistration artifacts. Lastly, while the association between Rad-Score and MRS was statistically significant, the correlation was modest. This initial observation may be attributed to tumor heterogeneity not fully resolved by the limitation of voxel size of 12 × 12 × 12 mm^3^ on MRS. Additionally, radiomics features and MRS metabolites may non-invasively capture the different aspects of tumor nature, while radiomics quantifies morphological and textural patterns and MRS offers complementary metabolic information. Besides, inherent technical limitations in both radiomics and MRS can introduce variability. Despite this, the statistically significant association between Rad-Score and MRS and further high-resolution tissue analysis shed light on a potential biological link between radiomic features and metabolic alterations within the tumor microenvironment. This finding could enlighten future risk stratification models by integrating radiomics and biological data.

## Conclusion

By developing a radiomic risk score using routine DW MRI, comparable with clinical standard ESMO classification, and establishing its association with increased choline metabolism, this study shows promising translational relevance in providing a personalized approach to therapy for high-risk EC patients.

### Supplementary Information

Supplementary Material 1

## Data Availability

Data generated or analyzed during the study are available from the corresponding author by request.

## Abbreviations

EC: Endometrial cancer
ESMO: European Society for Medical Oncology
FIGO: International Federation of Gynecology and Obstetrics
MRI: Magnetic resonance imaging
DWI: Diffusion-weighted imaging
MRS: Magnetic resonance spectroscopy
