# Supplementary material for: Endometrial cancer risk stratification using MRI radiomics: corroborating with choline metabolism
Source: Cancer Imaging. 2024 Aug 24;24:112. doi: 10.1186/s40644-024-00756-x (PMC11344325; doi:10.1186/s40644-024-00756-x)
Supplement: Supplementary file 1 — Supplementary Material 1 [file 40644_2024_756_MOESM1_ESM.docx]

## Supplemental Material

## Imaging protocol: DWI and MR spectroscopy

The magnetic resonance imaging (MRI) study were done on a 3 Tesla MR imaging unit (Skyra, Siemens, Erlangen, Germany) with both spine and body-phased array coils to cover the entire pelvis in the supine position. The parameters for diffusion weighted imaging (DWI) were as follows: single-shot echo-planar technique with fat suppression; TR/TE, 3300/79; average, 4; b-value = 0 and 1000 sec/mm^2^, section thickness, 4 mm; gap, 1 mm; matrix, 128 x 128; FOV, 20 cm; T2-weighted: 5630/87; average, 3; matrix, 256 x 320; FOV, 20 cm). For magnetic resonance spectroscopy (MRS), triplane localizer 1D MRS with point-resolved spectroscopy was used with the following parameters for PRESS: TR/TE, 2000 ms/35 ms; 128 averages; vector size, 1024 points; bandwidth, 1200 Hz; water suppression, selective band inversion with gradient dephasing. Also, non-water suppressed spectra were displayed as concentration references, four averages, with a total scan time of 6 minutes. MRS Data were analyzed using the LCModel software (version 6.3–0 K; Provencher, Ontario, CA, Canada) on a Linux workstation, which applied a linear combination of multiple spectra defined on the "Tumor" basis by generating a Gaussian peak between a minimum and expected linewidth for each simulated peak, then applying a Lorentzian line-broadening to them all (LCModel User's Manual). The Cramer-Rao lower bound (CRLB)value, which accounts for both line width and signal-to-noise ratio, was calculated to estimate the error in metabolite quantification. MR spectra were excluded if the CRLB exceeded 20% for choline (δ 3.2 ppm).

## Image processing and segmentation

ADC maps were generated using a monoexponential decay model with a b value of 0 and 1000 s/mm^2^ (VB17a; Siemens Trio TIM). Anonymous data were exported to an offline, personal computer. Two radiologists with 6 (Y.L) and 15 (Y.L.H) years of experience in gynecological radiology interpreted MR images independently blinded to clinical and histological information. The discrepancies were resolved by joining a third reader (G.L) for consensus. A consensus was made for the final analysis. Using an in-house developed software based on Matlab (Mathworks, Natick, Mass), region of interest (ROI) was drawn around the tumor on the ADC map with reference to the high b-value DW and T2-weighted images to delineate the whole tumor volume to minimize the slice selection bias. ROI was placed carefully to avoid contaminating the adjacent normal endometrium or areas of fluid.

## Radiomics analysis

In this study, MRI scans were normalized and first-order and 3D shape-based order features were extracted for radiomics analysis. Statistics of the median, standard deviation, skewness, and kurtosis were calculated from the feature responses of all voxels within the volume of interest (ROI). Redundant features were eliminated using the least absolute shrinkage and selection operator (LASSO) logistic regression model, and a radiomic score model (Rad-Score) was built using the selected features. A combined model (Rad-Signature) was also constructed with Rad-Score and clinical parameters including age, histopathology, and tumor grade. The models were tested in an independent dataset.

## Supplementary Table 1. List of features.

| *Category* | *Features* |
| --- | --- |
| First-Order (19) | Energy* |
|  | Total Energy* |
|  | Entropy |
|  | Interquartile Range |
|  | Kurtosis |
|  | Maximum* |
|  | Mean Absolute Deviation |
|  | Mean |
|  | Median* |
|  | Minimum* |
|  | Percentile 10^th^ |
|  | Percentile 90^th^ |
|  | Range |
|  | Robust Mean Absolute Deviation |
|  | Root Mean Squared* |
|  | Skewness* |
|  | Standard Deviation |
|  | Uniformity* |
|  | Variance* |
| Shape (17) | Compactness1 |
|  | Compactness2* |
|  | Elongation |
|  | Flatness* |
|  | Least Axis Length |
|  | Major Axis Length |
|  | Maximum 2D Diameter Column* |
|  | Maximum 2D Diameter Row* |
|  | Maximum 2D Diameter Slice |
|  | Maximum 3D Diameter |
|  | Mesh Volume |
|  | Minor Axis Length* |
|  | Spherical Disproportion |
|  | Sphericity |
|  | Surface Area* |
|  | Surface Volume Ratio* |
|  | Voxel Volume |

Note.—* represents features that were retained for constructing the radiomics model following the application of LASSO (Least Absolute Shrinkage and Selection Operator) regression.

## Supplementary Table 2: Correlation of radiomics features with MRS-derived choline levels and associated tissue metabolite concentrations.

|  | *MRS* | *Tissue NMR* |  |  |
| --- | --- | --- | --- | --- |
|  | Choline level | Choline | Phosphocholine | Glycero-3-phosphocholine |
| *Feature* | *P* value | *P* value | *P* value | *P* value |
| Robust Mean Absolute Deviation | .195 | .135 | .015* | .126 |
| Skewness | .015* | .551 | .565 | .076 |
| Minor Axis Length | .004* | .265 | .551 | .101 |
| Surface Area | .080 | .421 | .502 | .452 |
| Energy | .167 | .395 | .408 | .615 |
| Maximum | .915 | .055 | .004* | .209 |
| Maximum2DDiameterColumn | .008* | .731 | .979 | .033* |
| Total Energy | .167 | .395 | .408 | .615 |
| Minimum | .055 | .886 | .306 | .117 |
| Surface Volume Ratio | .080 | .790 | .157 | .309 |
| Uniformity | .637 | .320 | .391 | .832 |
| Maximum2DDiameterRow | .014* | .117 | .817 | .344 |
| Flatness | .195 | .693 | .798 | .553 |
| Median | .010* | .035* | .038* | .012* |
| Compactness2 | .470 | .259 | .891 | .528 |
| Root Mean Squared self | .012* | .034* | .012* | .009* |
| Variance | .457 | .125 | .015* | .009* |

Note.—* represents statistical significance by Pearson correlation analysis. MRS=magnetic resonance spectroscopy. NMR=nuclear magnetic resonance.
